# Overcoming Fear, Uncertainty, and Doubt: Artificial Intelligence (AI) and the Value of Trust

**DOI:** 10.7759/cureus.45576

**Published:** 2023-09-19

**Authors:** Erwin John T Carpio

**Affiliations:** 1 Radiology, Philippine College of Radiology (PCR), Quezon City, PHL

**Keywords:** opportunities and concerns, ai in medical imaging, ai and robotics in healthcare, ethics in ai, ethical implications of ai and robotics in healthcare

## Abstract

This is an editorial based on personal experience dealing with the fear, uncertainty, and doubt regarding artificial intelligence (AI) and radiology (my field of specialization). In the end, the most important tools to engage with these are education, research, and policy or regulation with the ultimate goal of forging trust, not just in the AI but also in the people that help make this technology possible.

## Editorial

When pushing for progress, the great wall against innovation is made of fear, uncertainty, and doubt. However, the greatest harm can sometimes come from the best of intentions.

When I search the web about artificial intelligence (AI) in healthcare, it typically falls under two extremes: the ridiculous hype train promising (or fearmongering) artificial general intelligence (when current AI only has the capacity for correlation but not necessarily causation) or the extremely regulated type of AI where you wonder how any worthwhile startup or endeavor could ever hope to get off the ground. Amidst the turmoil of the AI landscape, only the strongest, well funded, and most determined could possibly succeed in going beyond the hype and providing what is promised, without risking undue harm to patients. These large companies are at the cutting edge of AI. However, the thrust of such companies is usually horizontal (spanning various industries with large foundational models). What about the vertical, AI that drills down to the most specific use cases, particularly in healthcare?

With AI in healthcare, what usually comes to mind is big data, big companies, and big investments usually from the more advanced countries around the world competing on who edges out as the next big thing in AI and who leads as the driving force in AI’s future. However, AI is destined to derive from or affect not only the big picture but also the small picture. There is a huge resource-driven push for AI at scale but far less for AI in focus, how AI affects the larger whole and far less on how it affects the individual (or even marginalized). This is often pointed out by many ethical papers on AI. It is easy to say that AI can brilliantly fail only 1% of the time, but that becomes harder to swallow if that 1% was someone you know, such as your father, your mother, your sister, your wife, or your own child.

I would like to write about AI in focus and my experiences as a radiologist with small data and with little or far less money, writing Python and JavaScript, deploying a basic AI web application, and eventually writing potential AI policy in a small country, because AI is for everyone, big, small, and everything in between, and nothing big can ever truly stand without the small, the subtle components that comprise the sum of its parts.

A little personal history

I am a radiologist by profession with no conventional background in computer science. My interest was originally in web development. I wanted a new skill or a hobby. It was suggested that I learn a new language such as French or Japanese. I chose JavaScript. I studied at a free online university. I learned to build websites from scratch and use various application programming interfaces (APIs) and integrated development environments (IDEs) and even coded a tic-tac-toe game “bot” (my first try in developing “computer intelligence”), and it was fun. Then, I saw Geoff Hinton on YouTube [1]. It was a shocking revelation. I heard that we should “stop training radiologists now” and that AI will be better in the near future. These were unforgettable words from one of the godfathers of modern-day AI. I had already heard of AI before this. I was slowly learning Python, but I had no idea that my position in healthcare was already on shaky or even nonexistent ground.

Fear

The usual fear then and even now is, will AI take my job away? Am I obsolete? AI can allegedly drastically “learn” within a few hours, days, or even weeks what would take a radiologist to learn in years (so long as it had way more data). However, I thought that if AI can learn what I do in a short amount of time, how much time or effort would it take for a human mind (natural intelligence) to learn, develop, and deploy AI with less “data”? I took free courses online. I joined a hackathon and built and deployed my first web application called RadLens, and it was eventually featured in the Google TensorFlow Dev Summit in 2020 [2,3] and the TensorFlow developer blog [3]. It was an exhilarating experience. Then, I was invited to the Philippine College of Radiology as a member of the AI subcommittee. After all this, what I realized was that the true question was not “will AI replace me?” but is AI safe for our patients? Is it safe for our radiologists? Is it safe for the “human” practice of medicine? These were and still are troubling questions.

The sales pitch to a radiologist is usually that we (the rads or radiologists) have nothing to fear. AI will not replace us. It is only there to help us. Rads will still sign out the final report. This however can also be taken another way. They (“the AI vendor or developer”) have nothing to fear since no matter how many times their AI makes a mistake, the radiologist has to correct it. There is absolutely no legal liability on the side of the vendor. However, the one time the AI is correct and the radiologist is the one at fault, there is immediate legal fallout. All the risk is taken upon by the radiologist. To mitigate such risk, will the radiologist just choose to side with whatever the AI says? This leads to automation bias. If the radiologists start doing only what the AI says to minimize legal repercussions, then would not that truly make the radiologist even more replaceable? Furthermore, without a human “brave” enough to actively correct AI, how will this affect the patient?

Reporting guidelines for adverse incidents with regard to software as a medical device are slowly being released from countries such as the United Kingdom [4]. Some concern has been expressed on how it would be difficult for AI startups to take on the ever-growing responsibility with regard to AI. However, in healthcare, responsibility and liability are a must especially when dealing with patients’ lives. An area under the curve (AUC) of 0.7-0.8 is not just a set of numbers to be min-maxed. The 0.3 or 0.2 that the AI is misdiagnosing is not just a statistic. These numbers have names, names that belong to real people. Some doctors go through a stage of what is classically called the “god syndrome” where they feel empowered by knowledge over the human body. Most medical students are the best of the best from their graduating classes, which may add to the feeling of superiority. However, real-life medicine breaks all our egos down in the end with a heavy dose of reality. It is not about the last A+ or 90+ you got on the last written examination (a human neural network’s “performance” metric test). It is about the smile on your last patient’s face (even if all you did was lend an ear during the most difficult stage of their disease). It is about taking in the brunt of a family member’s frustration when treatment is not working (even if there is an acceptable 0.2-0.3 chance of failure). Healthcare is not about min-maxing numbers on a laboratory report. It is about helping people at their most difficult times and knowing how your actions can drastically affect another person’s entire life. An acceptable machine score of 0.7-0.8 becomes far less “certain” in the context of an individual life.

Uncertainty

I recently finished the Radiological Society of North America (RSNA) Imaging AI Advanced Certificate course, and one of the key phrases I picked up from the lectures was that it is not just a matter of metrics. It is a matter of trust. The AI will do what it is programmed to do, which is min-max a pattern matching algorithm given the data. What we really need to ask is not can we trust the AI but can we trust the data. At the end of the day, it is still a computer, garbage in, garbage out. Whether intentionally or unintentionally, if the data fed into the AI had some unwanted bias (based on age, sex, or any other unforeseen trait), there is a chance that the AI will learn that bias and apply it.

Big data is often the answer to mitigate the problems with bias. Even more than big data is good data, data that is properly curated by domain experts (radiologists) and responsibly handled by data scientists to maximize proper representation even of marginalized groups and to minimize the likelihood of unwanted bias. However, how is this “golden data,” this new invaluable resource, gathered? There is a thin line between data gathering for the common good and data mining for profit. In advanced countries, there are huge open-source data sets for the common good and quite a large plethora of online platforms and services that allow for the steady and consistent growth of radiologists in the field of AI as “domain experts,” which I believe is the natural evolution of radiology in the inevitable landscape of AI. Such amazing work can hopefully be applied to and help the underserved patients in the developing nations of the world in the fight against the most pervasive health problems today, one of which in particular is pulmonary tuberculosis. The potential of this technology excites me as both a radiologist and AI enthusiast. However, I do not live in the more advanced countries trying generously to share and apply AI to the problems of the world. I live in a small country, one of those hoping to receive the benefits of AI in healthcare. As stated earlier, sometimes, the best of intentions (if unregulated) may lead to harm.

Current AI has the potential to change and improve the world of healthcare by drastically solving the lack of expertise in underserved areas. The power of AI is its potential to solve “narrow problems” at scale. I think that this is where the problem begins. There is a need to highlight that what we have is artificial “narrow” intelligence and not “general intelligence.” Just as easily as AI can solve a narrow problem, so too can it mass produce or magnify a tiny problem of bias at scale. There is a generous outpour of foreign AI software in my country, and there is an equally eager, if not emergent, need to receive such technology to aid in our mitigation of disease such as pulmonary tuberculosis. We receive amazing studies from abroad with AI garnering amazing performance metrics reaching, if not exceeding, those of a human radiologist. However, as I have learned from my recent courses in AI both for radiology and in Python and JavaScript, AI has a problem: covariate shift.

Covariate shift is when a number of factors change such that the performance of the AI model can be affected. Examples include the distribution of disease within the population or technical factors in acquiring the diagnostic image. You can have an AI model exhibit amazing performance metrics at one hospital and then have significantly decreased performance if taken to another hospital because of a slight difference in the imaging protocol or in the radiologic equipment used for the procedure. In terms of population demographics, an AI model can have amazing detection rates for a population with a high incidence of the disease being screened for but fare far less favorably in a population where the disease has a lower incidence. This emphasizes the need for the local and external validation of AI models in healthcare not just for the general population but also for the specific site, clinic, or hospital where it will be used. Even the WHO guidelines for the use of AI in pulmonary tuberculosis classify its own recommendations as conditional with low certainty of evidence [5] and give an entire list of caveats on the usage of AI ranging from the target populations to model performance, to even the methods for choosing a threshold score for AI.

Foreign performance studies for the general population are necessary, but it cannot end there. There has been a pressing need to educate in my country regarding the importance of external local validation to minimize the unwanted effects of AI-related bias and covariate shift. This is in no way the fault of those offering AI to help and in no way the fault of those eager to accept aid. Again, it is just a matter of minimizing the chance of good intentions leading to unwanted harm. This is best done through education, which is a slow and steady process that sometimes simply cannot compete with the speed of the AI hype train that is currently the driving force of uncertainty and the next part of this editorial.

Doubt

AI will not totally replace humans in the field of radiology. However, the practice of radiology will change and evolve into a form that is yet to be defined, as AI takes on more correlational roles and humans switch to more cognitive ones. I would like to believe that in my country, we too can achieve the current niche of radiology that is happening in more advanced nations: the radiologist as a “domain expert,” deep into AI research, publishing groundbreaking papers, and presenting at conventions for the benefit of all and the common good. However, something else seems to be happening. There is another potential role or niche current radiologists can fill in the AI landscape: the “manual labeler.” Radiologists can be hired to manually label radiologic images by third-party companies that provide labeling services for the actual AI developers that need such services. The process is streamlined, efficient, and cost-effective. However, the radiologist in this scenario is disaggregated from the center of AI research and instead becomes a simple cog in the large machinery that is AI research and development for healthcare. In this scenario, my current limited feedback from radiologists is doubt. There is doubt if radiologists still have any future in the light of AI.

Another concern of being cornered into the niche of manual labeler is the explosion of generative AI. If labeling medical image data becomes the main niche of future radiologists, how will that compete with further advancements when generative AI can be asked to produce 10,000 images of subdural hematoma and 10,000 images of epidural hematoma to help train another discriminatory AI classifier? Again, the solution to this is education. Fortunately, there are already a number of AI-related radiologic studies in my country that can help foster the role of the radiologist as the domain expert. I trust in the ingenuity of my fellow radiologists and hope that our niche will still fall into the category of domain expert.

There are ethical questions under all of this doubt. Should radiologists accept jobs that let them trade in their domain knowledge to train AI for a fee and nothing more? Should radiologists accept a role in AI development that is purely contractual and not academic, a role wherein they relinquish all rights to or responsibility over any AI that might be produced from their efforts, AI that might eventually be used to help the radiology workflow in the best-case scenario or replace other radiologists or even themselves (a less appealing outcome)? Radiologists labeling for AI is nothing new. Some of the greatest advancements in AI for healthcare have been made possible with open-source data sets, and I believe that this is the ideal scenario. Open-source data sets are essentially produced for the common good and are available for all responsible enough to use the data. The question however is for siloed data sets and siloed AI models from private companies. Who has the right to truly “own” healthcare data? Furthermore, who truly has the right to profit from such data? Should private companies not be allowed to keep siloed data sets and AI models? How can they be sustainable or even profitable if they do not secure their technological advantage? Although open-source data is a valid resource for AI development, so too is private investment. Beyond data and investment, however, we must also consider the needs of the patient. The definition of the “common good” becomes blurred as various interests enter the fray. What role should the radiologist play in all of this? Which role is “good” or “bad?” In the end, it may not even be about “good” or “bad” but simply “consequences.” There can be considerable doubt in the future of radiology, but the degree of doubt is coupled with the choices we make today, because the role we pick today determines our niche as radiologists tomorrow.

Trust

I was once asked by a fellow doctor, “does it matter if we completely understand how AI works so long as it does?” Do we fully understand how a car works before we can drive it? This was also tackled in some of my AI courses for radiology. One of the conclusions I gathered, even if there are circumstances wherein we may no longer need to fully understand the intricacies of the deep neural network (what architecture was used, what activation functions were employed, how many epochs or batches, or even what back propagation or stochastic gradient descent even means), there is still the need to develop trust. However, we can only trust machines to min-max data. Can we trust the “data” itself and data that can shift? I would like to add another layer to this concept of trust. We can argue about trusting AI or the data behind it, but I would like to believe that I can and should be able to trust “people.” This is where policy and regulation come in. I have enjoyed and continue to enjoy writing code for AI, but on my humble journey on this truly remarkable path, I have also begun to focus on helping to write policy.

I have realized that in AI for healthcare, we cannot just write code for machines; we also need to help write policy for people. Policy can range all the way from the developer’s end by determining the proper performance metrics (area under the receiver operating characteristic {AUROC} or area under the precision-recall curve {AUPRC}) and threshold score for AI under the larger theme of AI model maintenance all the way to the end user for whom the scope and limitation of the AI should first be made clear via proper education and training. Anyone can wield a scalpel, but it takes an educated and well-trained surgeon to properly use it. The surgeon also does not use surgical equipment from just anyone. He must trust that those preparing his instruments followed the proper standards for healthcare. AI can serve as the new razor of the radiologist as he surgically dissects medical imaging data. This brings me to one of the more important principles I have learned when helping to write policy for AI: the principle of “intended use.” I must reiterate that what we have now is more of artificial “narrow” intelligence instead of artificial “general” intelligence. Current AI can solve very narrow specific problems at scale, which is the technology’s greatest potential. This is also why it is important to clearly define the specific problem being solved before applying AI. We cannot just deploy AI into a healthcare facility or radiology workflow without clearly defining its role, scope, and limitations, as well as the contingencies needed to help mitigate unwanted outcomes. Just as important as knowing when to use AI is knowing when not to use AI.

In the end, it is not a question of trusting just the studies proving the performance of AI. It is about trusting the people behind the AI, trusting that they are doing their best to consistently update and ensure that the performance of the AI model delivers on the ultimate goal of AI in healthcare, which is not to get an AUC of 0.8-0.9 but to help improve the patient’s life. This trust is forged when we share a common standard of policy.

The great walls against innovation are fear, uncertainty, and doubt. Fear is not necessarily a bad thing. It keeps us grounded and protects us from venturing into unknown danger. To break through the fear, we must carve a gateway through that wall with education and research. However, a gateway once made can still collapse. We must build arches or infrastructure, a platform around this gateway that people can feel safe walking through without the roof falling on our heads and a platform that allows for proper maintenance and improvement of the gateway. A gateway also needs a door, or else, anyone can just pass through. AI is a powerful technology, but it is also a neutral technology. Its propensity to aid or hurt depends on the people who use it. The door should be designed to let the right people in and keep the wrong people out. This is why policy and regulation are just as important as the AI itself. Finally, the only way to test this gateway is to walk through it, and what better way to remove fear, uncertainty, and doubt than to walk through it together, with people we trust.
